# Impact of Intensive Physiotherapy on an Adolescent with Severe Genu Valgum Deformities: A Case Report

**DOI:** 10.7759/cureus.33907

**Published:** 2023-01-18

**Authors:** Ayush S Agrawal, Pratik Phansopkar

**Affiliations:** 1 Musculoskeletal Physiotherapy, Ravi Nair Physiotherapy College, Datta Meghe Institute of Medical Sciences, Wardha, IND

**Keywords:** quality of life, electrotherapy, physiotherapy, gait training, genu valgum

## Abstract

The genu valgum is one of the anomalies of the lower extremities in the coronal plane (knocked knees). Children might be vulnerable to it as well. It may also be hereditary, with females being more likely to develop it. A 14-year-old female patient had severe genu valgum anomalies of the bilateral lower extremities. The patient is in pain, unable to stand, and sit on the ground. On the numerical pain rating scale (NPRS), the level of pain was rated as 8/10 while activity and 5/10 during rest. The patient received physiotherapy treatment for a total of six weeks, which included exercises, electrotherapy, and gait training. As a result, the patient experienced a reduction in pain, which was rated 2/10 while activity and 1/10 during rest, and an improvement in range of motion, and functional activities. Correcting the gait, enhancing the quality of life, and restoring functional ability are all significantly impacted by physiotherapy. Based on the current case study, conventional physical therapy improved functional goals gradually and significantly, which in turn potentially aided in a successful recovery.

## Introduction

One of the biggest and most intricate joints in the human body is the knee. The knee connects the shin bone (tibia) to the proximal end of the thigh bone (femur) [1]. Genu valgum is most usually noted as a knock-knee [2]. Genu valgum is one of the lower extremity anomalies in the coronal plane (knocked knees) [3]. The most prevalent cause of genu valgum is idiopathy. However, in developing and third-world countries, genu valgum is common [1]. Rapid physical therapy results in the accomplishment of functional objectives. When the legs are straightened, the knees in genu valgum (knock-knee) are bent toward the body's midline and come into contact with one another [4]. Genu valgum is a cosmetic deformity that can also change gait by repetitively contacting knees during jogging. It alters the Q angle and increases the risk of dislocation of the patella. Weight-bearing moves to the medial side of the foot, producing the appearance of a flat foot and necessitating regular footwear replacement [5]. In addition to being unable to walk or sit up, the patient had been suffering from severe discomfort. Due to knee-knocking, lack of foot clearance, and pre-swing phase, the gait pattern was impacted [6]. Early physical therapy results in the accomplishment of functional objectives. In these situations, physiotherapy focuses primarily on correcting the malformation. For six weeks, the patient underwent physiotherapy, which comprised exercises, electrotherapy, and gait training. As a consequence, the patient's discomfort, range of motion, and functional activity improved.

## Case presentation

A 14-year-old female presented with severe genu valgum deformities of the bilateral lower extremities and was associated with an uncoordinated gait. After consulting with an orthopedic surgeon, the patient was managed with supracondylar osteotomy of the right femur with plate fixation three years back. In March 2022, the patient underwent other surgical treatment of the left lower limb managed by lateral wedge osteotomy followed by hip spica cast application which was kept for 1.5 months. After that, the patient started physiotherapy.

Radiological findings

On Inspection

**Figure 1:**
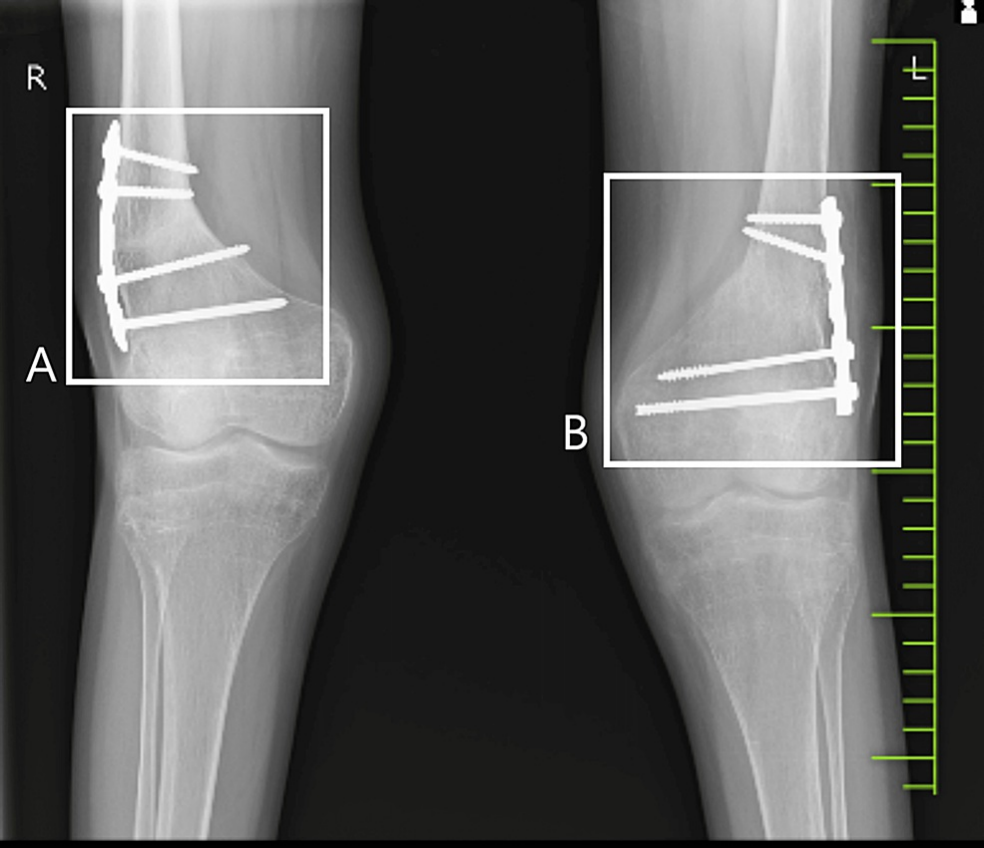
Post-operative (32 days) radiological findings. Box A shows the right knee; Box B shows the left knee.

After getting the full permission of the patient's parents, a thorough physical examination was performed (Figure 1). The patient was examined in a supine and standing position. The attitude of patient was apprehensive. The hip and knee joints were in a 70-degree flexion position. Diffuse swelling was seen over the right knee joint. Skin appears tense and shiny. Healed surgical scars were extending from the distal thigh centering over the patella seen in the previous surgery. Muscle wasting was seen over the right and left thigh. On the numerical pain rating scale (NPRS), the intensity of pain was rated as 8/10 while exercising and 5/10 during rest.

On Palpation

On palpation, inspection findings were confirmed. The temperature was normal. Grade 2 tenderness was present over the joint line. The patient is unable to extend her left leg. Dorsalis pedis and posterior tibial artery pulsation were present. Soft tissue tightness in the muscles and ligaments in both upper limbs was NIL. Severe posterior knee capsule tightness in the right knee was discovered during the lower limb soft tissue tightness evaluation by actively performing a range of motion or giving resistance. By evaluating of manual muscle testing, it shows restrictions of normal functional activity.

Manual Muscle Testing

Bilateral upper limb strength was assessed for muscular strength and found to be 4/5 on the standard grading scale. On an Oxford grading system for muscular strength, the bilateral lower limb strength was discovered to be 3+/5.

Range of Motion

Both upper limbs had a full and usable range of motion at the shoulder, elbow, wrist, hand, and fingers (complete and pain-free). Tables 1, 2 show the pre- and post-rehabilitation range of motion for the lower limbs.

**Table 1:** Pre-rehabilitation range of motion

Joint	Movement	Right	Left
Hip	Flexion	0-110	0-100
	Extension	0-15	0-10
Knee	Flexion	0-110	0-125
	Extension	110-0	110-0
Ankle	Dorsiflexion	0-30	0-30
	Plantarflexion	0-25	0-25

**Table 2:** Post-rehabilitation range of motion

Joint	Movement	Right	Left
Hip	Flexion	0-130	0-130
	Extension	0-15	0-12
Knee	Flexion	0-130	0-135
	Extension	130-0	135-0
Ankle	Dorsiflexion	0-10	0-10
	Plantarflexion	0-50	0-50

Physical therapy management

We assessed the patient's strength, range of motion, tightness, and discomfort on the first day of treatment. The patient received information about the surgical technique before therapy began. The patient has emphasized the value of exercise for his speedy return to ADLs and improved rehabilitation. Gait training was given to the patient and ergonomic advice was suggested which included posture correction. Table 3 has further management.

**Table 3:** Physiotherapy management SLR: Straight leg raise; ROM: Range of motion

phase	Physiotherapy regimen
Phase 1 Immediate post-operative (3-5 days)	To reduce pain -electrotherapeutic modality + Relaxation Training. To reduce inflammation-cryotherapy. To reduce swelling -speedy quadricep settings or electrical stimulation under pressure bandage with limb elevation; resistive ankle, foot movements, and SLR. To prevent reflex inhibition-sustain frequent isolated quadriceps setting withhold for 6-10 seconds. Supported relaxed knee passive swinging in a small range with the normal collateral leg.
Phase 2 Early healing (5-15 days)	Gradual but definite progression of early measures in phase 1 along with Knee ratchet pedocycle or static exercise regimen, Weight transfers Supported or full weight-bearing ambulation Knee ROM should be around 120 degrees
Phase 3 Late healing (15-21 days)	Vigorous progressive resistive quadriceps exercise supported and guided functional positions. Floor squatting, cross leg-sitting, and prone heel sitting (kneeling). Standing on the affected leg alone, ambulation unsupported or with minimal support, but no limp. Knee ROM should be around 120 degrees
Phase 4 Conditioning (3-5 weeks)	High sitting, speedy isotonic full ROM, relaxed knee swinging. Progressive resistive quadriceps exercises. Balance activities – proprioception. Gait training. Return to work
Phase 5 Functional progression(6 weeks onwards)	Spot running – by holding wall bars. Straight jogging. Straight running. Straight hopping. Agility drills (e.g. figure-of-eight running). Gradual return to sports

## Discussion

A common orthopedic condition in children is genu valgum [7]. The majority of these individuals come to the hospital for a cosmetic issue. The majority of these patients with Physiological Genu Varum and Physiological Genu Valgum progress through the typical developmental phases [6]. It can be genetic with a higher incidence in females. Many surgeries have proven effective but are also associated with physiotherapy management which deals with rehabilitation [8]. The standard treatment for these patients is electrical modalities with cryotherapy, stretching, mobilization, strengthening, and open and close chain exercises. Electrical modalities include interferential therapy, transcutaneous electrical nerve stimulation, and ultrasound. Physiotherapy treatment goals depend upon the patient's primary problem and on deformity correction. Various manual therapy techniques are also used for deformity correction, and strengthening is given to a specific group of muscles to maintain muscle strength. While certain muscles seem to become tighter, others deteriorate with time. So, the property of each muscle is identified and treated accordingly. It helps significantly in pain management which leads to gaining confidence in patients. The main goal of the physical therapy intervention was to improve the range of motion and keep the muscles strong and healthy for walking. Cryotherapy has significantly reduced discomfort, encouraging patients to exert more significant efforts [9]. Nutritional rickets and trauma are the primary causes of genu valgum deformity in industrialized and developing nations [10]. An irregular gait, functional issues, early onset of arthritis, anterior knee discomfort, patellar maltracking leading to patella-femoral dislocation, and trouble running are all caused by persistent genu valgum [11]. Different distal femoral osteotomies have been documented in the literature for the correction of valgus alignment in late adolescents and young adults. The purpose of therapy is to restore proper limb alignment and stop further joint deterioration.

## Conclusions

Early identification of the deformity and providing the correct management for the same is necessary. Physiotherapy has a significant effect in correcting the gait and improving the quality of life and functional ability. The above case report suggests that classic and prompt structural physical rehabilitation led to improving the functional goals progressively and significantly which majorly leads to successful recovery. The patient showed great cooperation during the intervention period and now the subject is able to walk with the support of a walker. According to the case study, classic and speedy structural physical rehabilitation improved gait and quality of life.
